# Editorial: The association between oral health and mental health

**DOI:** 10.3389/froh.2025.1610146

**Published:** 2025-06-05

**Authors:** Jing Kang, Huabin Luo, Jianhua Wu

**Affiliations:** ^1^ Centre for Clinical Translational Science, Faculty of Dentistry, Oral and Craniofacial Sciences, King’s College London, London, United Kingdom; ^2^ Department of Public Health, East Carolina University, Greenville, NC, United States; ^3^ Centre for Primary Care, Wolfson Institute of Population Health, Barts and the London School of Medicine and Dentistry, Queen Mary University of London, London, United Kingdom

**Keywords:** oral health, mental health, depression, anxiety, oral disease

**Editorial on the Research Topic**
The association between oral health and mental health

Mental health disorders—including depression, anxiety, and stress-related conditions—pose a mounting global challenge (1). Affecting hundreds of millions worldwide, they contributed significantly to disability and quality of life (2). Yet, one often overlooked facet of this burden is its intricate and bidirectional relationship with oral health (3).

Oral disease, ranging from dental caries and periodontal diseases to tooth loss, are among the most prevalent chronic conditions globally (4). Individuals with mental health disorders are disproportionately impacted by poor oral health, with contributing factors including poor hygiene, side effects of psychotropic medications (such as xerostomia), lifestyle factors like smoking, and barriers to accessing timely dental care (5, 6). The relationship also works in reverse: poor oral health can exacerbate mental health conditions by contributing to chronic pain, social stigma, impaired self-esteem, and reduced social participation (7).

In this context, our research topic called for studies examining the association between oral and mental health. We called for studies investigating not only the association between these domains but also underlying mechanisms, shared risk factors, and opportunities for integrated care.

For instance, Powell and Taylor examined data from the 2019 Medical Expenditure Panel Survey in the U.S. and found that self-reported poor mental health was significantly associated with complete tooth loss (edentulism). Despite an overall edentulism prevalence of 6%, rates were markedly higher among those with poor mental health, particularly in populations affected by smoking and lower educational attainment. These findings highlight the importance of considering oral health in public mental health strategies—and vice versa.

Cebrino and Portero de la Cruz analysed depression risk factors in over 25,000 Spanish adults with oral health issues. Depression prevalence stood at 7.81%, with notable gender disparities: 10.14% in women vs. 5.39% in men. The study illuminated gendered patterns in oral hygiene attitude and disease burden, with women more likely to have prosthetics and routine checkups, and men more prone to caries and extractions. This highlights the value of gender-sensitive, integrated care approaches.

Joury et al. proposed an innovative syndemic framework—where mental, physical, and oral health problems cluster and reinforce one another under conditions of social adversity. Drawing on diverse datasets including UK Biobank and NHANES, they aimed to explore these interactions using structural equation modelling. This approach opens new doors to understanding the social determinants of intertwined health outcomes, and the structural interventions needed to address them.

Two papers addressed mental health in people born with cleft conditions. Xia et al. critically reviewed the current use of patient-reported outcome measures (PROMs) for complications such as velopharyngeal insufficiency and sleep-disordered breathing. They called for cleft-specific PROMs to ensure truly patient-centred care. Yang et al.’s case-control study showed that individuals with cleft lip report higher appearance-related distress and greater anxiety and depression—independent of cleft type. This draws attention to the mental health burden of craniofacial differences, especially in cultures where appearance carries social weight.

Zhang et al. conducted a comprehensive meta-analysis examining the link between sensorineural hearing loss (SNHL) and mental health. Synthesising data from over 675,000 individuals, they confirmed a robust bidirectional relationship between SNHL and both depression and anxiety. The association varied by age, geography, and diagnostic criteria. These findings reinforce the broader theme that sensory and oral health are critical components of mental well-being.

Collectively, the contributions to this research topic reinforce the message that oral and mental health are deeply interconnected and their association should be further explored via various aspects in oral and mental health. Whether through syndemics of adversity, cleft-related distress, or tooth loss-associated depression, the evidence is clear (thanks to the unique message provided by each contributing publication in this research topic): we need more integrated, interdisciplinary approaches to healthcare. Oral health should no longer be siloed from mental health services but recognised as a vital determinant of psychological well-being.

Looking ahead, future research should prioritise:

• Preventive strategies that address both oral and mental health risks in tandem
• Interventions tailored for vulnerable groups, including those with congenital conditions or chronic comorbidities
• Policies that integrate dental and mental health services within primary care—such as mental health screenings in dental practices for high-risk patients or referral pathways from mental health facilities based on patients’ self-reported oral health—can support more effective prevention services.

It is time to move beyond the artificial divide between mind and mouth, and further research is essential to deepen our understanding in this area.
